# Complication rates of transrectal and transperineal prostate fusion biopsies – is there a learning curve even in high volume interventional center?

**DOI:** 10.1590/S1677-5538.IBJU.2023.0054

**Published:** 2023-04-15

**Authors:** Guilherme Moratti Gilberto, Marcelo Froeder Arcuri, Priscila Mina Falsarella, Guilherme Cayres Mariotti, Pedro Lemos Alves Lemos, Rodrigo Gobbo Garcia

**Affiliations:** 1 Centro de Medicina Intervencionista Hospital Israelita Albert Einstein São Paulo SP Brasil Centro de Medicina Intervencionista, Hospital Israelita Albert Einstein. São Paulo. SP, Brasil

**Keywords:** Prostatic Neoplasms, complications [Subheading], Radiology, Interventional

## Abstract

**Purpose:**

To analyze the learning curve regarding complication rates of transrectal prostate biopsy (TRPB) versus transperineal prostate biopsy (TPPB), using real time software-based magnetic resonance imaging ultrasound (MRI-US) fusion techniques, along with first year experience of transperineal approach.

**Materials and Methods:**

retrospective unicentric cohort study at a quaternary care hospital. Medical records of all consecutive patients that underwent TPPB between March 2021 and February 2022, after the introduction of MRI-US fusion device, and those who underwent TRPB throughout the entire years of 2019 and 2020 were analyzed. All complications that occurred as consequences of the procedure were considered. Descriptive statistics, Chi-squared and Fisher tests were used to describe complications and compare the two groups.

**Results:**

A total of 283 patients were included in the transperineal group and 513 in the transrectal group. The analysis of a learning curve for the transperineal method showed lower complications rates comparing the first six months of TPPB procedures (group 1); The complication rate for TPPB was lower than that of TRPB (55.1% versus 81.9%, respectively; p<0.01). TPPB showed specifically lower rates of hematuria (48.8% versus 66.3%;p<0.001) and rectal bleeding(3.5% versus 18.1%; p<0.001). There were no cases of prostatitis after transperineal biopsies and three cases (0.6%) after transrectal procedures.

**Conclusions:**

We evidenced the learning curve for performing the transperineal biopsy, with a lower rate of complications for the experienced team, after 142 cases after 6 months of practice. The lower complication rate of TPPB and the absence of infectious prostatitis imply a safer procedure when compared to TRPB.

## INTRODUCTION

Prostate cancer is the second cause of mortality amongst men (1). The increase in prostate cancer screening with prostate specific antigen (PSA) and digital rectal exam (DRE), and the recent development of the superior accuracy of the multiparametric prostate magnetic resonance imaging (MRI) have led to early diagnosis of clinically significant cancers and consequently reduction in morbidity and mortality due to early treatment (2, 3).

Confirmatory diagnosis of prostate cancer is performed through biopsy, which may be associated with techniques that reduce false negatives, such as MRI/transrectal ultrasound (US) fusion targeted biopsy (4). However, using a transrectal approach in most parts of the World might cause elevated complications rates; some of them are potentially life-threatening, such as prostatitis, sepsis, and severe rectal bleeding (5).

Within this scenario, transperineal prostate biopsy (TPPB) has emerged as an alternative that overcomes some limitations of transrectal prostate biopsy (TRPB) and increases the safety profile of the prostate biopsy procedure (6). This method uses percutaneous access to the prostate through the perineum, without perforation of the rectum. Therefore, it is sterile and avoids the trajectory of the rectal arteries or hemorrhoidal plexus when properly performed.

However, literature has not described whether there is a learning curve related to the procedure. Would the inexperience of a team of interventional radiologists be a limiting factor in performing the procedure? The purpose of this study was to describe the initial learning curve of experience with TPPB MRI/US fusion device in a quaternary hospital and to compare the complication rates with those of previous routine TRPB at this institution. Its importance, in addition to demonstrating the learning curve, was its pioneering role in reporting the replicability of transperineal MRI fusion biopsy in a large-volume tertiary center in Latin America.

## MATERIALS AND METHODS

This was a retrospective unicentric cohort study performed in a large quaternary hospital. Medical records of all consecutive patients that underwent TPPB between March 2021 and February 2022 and those who underwent TRPB from January 2019 to December 2020 were reviewed. The inclusion criteria were patients who were referred to receive prostate biopsies for clinical suspicion of prostate cancer by the patient’s urologist including high PSA levels, abnormalities on digital rectal examination or prior imaging studies with a suspicious lesion. The exclusion criteria were incomplete medical records.

The study was approved by the institutional review board and performed in accordance with the Helsinki Declaration (CAAE: 60310822.6.0000.0071).

### Procedures

The indication of the prostate biopsy was based on clinical suspicion of prostate cancer by reference urologists (including high PSA levels, abnormalities on digital rectal examination and/or prior MRI with a suspicious lesion). The biopsy was contraindicated in case of a positive urine culture or increased bleeding risk (identified through international normalized ratio > 1.5, platelets < 50,000 x 109/L or use of anticoagulants).

According to the institution’s protocol for prostate biopsy, all patients underwent prophylactic antibiotic therapy with 2000mg of intravenous ceftriaxone. Patients underwent moderate sedation or general anesthesia depending on the anesthesiologists’ criteria. No additional anesthetic block was performed in the transrectal group, while in the transperineal group an anesthetic block of the prostatic plexus and of the pudendal nerve with a long-acting anesthetic (ropivacaine 0.75%) was performed.

**Figure 1:**
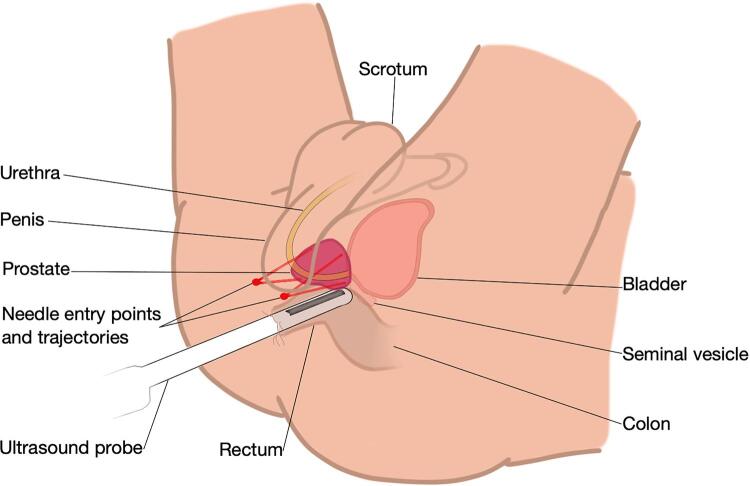
Schematic transperineal prostate biopsy: patient in lithotomy position and transrectal ultrasound is performed.

The transrectal procedures were performed by one out of 10 experienced interventional radiologists, with at least 10 years of TRPB and little TPPB experience. Transrectal ultrasound was performed with a GE Logic E9 device and biopsies with Acecut 18G needles (TSK Lab Jap.). Transperineal ultrasound was performed with Esaote My Lab and Canon Applio A. A freehand technique was used (grid was not used) (Figure-1).

Red dots demonstrate the skin puncture sites and red lines represent possible needle trajectories toward the prostate.

### Outcomes

The primary outcome was the learning curve using comparative rates of complication between TPPB and TRPB groups. All complications that occurred as consequence of the procedures were considered as described in the patient’s medical records according to the Clavien-Dindo classification (7). The complications of interest were hematuria, rectal bleeding, urinary retention, prostatitis, and pain with or without the need for analgesia. Less common or relevant complications, such as anesthetic complications, were named as “other”. For this analysis, the transperineal group was divided into biopsies performed by the inexperienced team (group 1), comprising the first six months (March 2021 to August 2021) of TPPB procedures; biopsies performed by the now experienced team composed of the same physicians (group 2), comprising the following six months of procedures (September 2021 to February 2022).

The secondary outcome was pathological results of prostate biopsies between TPPB and TRPB groups, which were measured by Gleason and ISUP classifications.

### Statistical considerations

There was no predefined sample size for this study, as all consecutive patients who underwent TPPB and TRPB in the predetermined periods were included.

Categorical variables were described with descriptive statistics as frequencies and percentages. Complication rates were compared by chi-squared and Fisher’s tests, when appropriate. The comparisons of pathological Gleason and ISUP reports, and complications were performed by Mann-Whitney test. p values < 0.05 were considered statistically significant.

## RESULTS

A total of 513 patients were screened for the TRPB group in 23 months (22.3 biopsies/month). The TPPB group included 283 patients (in 12 months – 23.5 biopsies/month): 142 biopsies were performed in the first 6 months (group 1) and 141 in the following 6 months (group 2).

The complication rate for the TPPB group was lower than that of the TRPB group (55.1% versus 81.9%, respectively; p<0.001). Complications such as hematuria, rectal bleeding and low-grade pain were also significantly lower for TPPB, as described in Table-1. Pain requiring analgesia, urinary retention, were lower in the TPPB group, but without statistical significance. There was no biopsy-associated prostatitis in the transperineal group, and three cases associated with transrectal biopsies. Two patients with infectious prostatitis required hospitalization for intravenous antibiotic therapy, and one of them was admitted to the intensive care unit. There were no deaths in either group.

**Table 1:** Complication rate between TPPB and TRPB and between TPPB groups 1 and 2.

Complication	Transperineal (N=283)	Transrectal (N=513)	p value
**All – no. (%)**	156 (55.1)	420 (81.9)	<0.001
**Hematuria – no. (%)**	138 (48.8)	340 (66.3)	<0.001
**Rectal bleeding – no. (%)**	10 (3.5)	93 (18.1)	<0.001
**Pain without need of analgesia – no. (%)**	12 (4.2)	48 (9.4)	0.009
**Pain requiring analgesia – no. (%)**	12 (4.2)	23 (4.5)	0.87
**Urinary retention – no. (%)**	3 (1.1)	12 (2.3)	0.20
**Prostatitis – no. (%)**	0	3 (0.6)	0.56
**Other – no. (%)**	7 (2.5)	15 (2.9)	0.71
Complication	TPPB Group 1 (N=142)	TPPB Group 2 (N=141)	p value
**All – no. (%)**	95 (66.9)	61 (43.3)	<0.001
**Hematuria – no. (%)**	84 (59.2)	54 (38.3)	<0.001
**Rectal bleeding – no. (%)**	9 (6.3)	1 (0.7)	0.02
**Pain without need of analgesia – no. (%)**	8 (5.6)	4 (2.8)	0.24
**Pain requiring analgesia – no. (%)**	8 (5.6)	4 (2.8)	0.24
**Urinary retention – no. (%)**	2 (1.4)	1 (0.7)	>0.99
**Prostatitis – no. (%)**	0	0	-
**Other – no. (%)**	3 (2.1)	4 (2.8)	0.712

TPPB = transperineal prostate biopsy; TRPB = transrectal prostate biopsy; no = number; % = percentage Group 1: TPPB y in the first 6 months (March to August 2021); Group 2: TPPB in the following 6 months (September 2021 to February 2022)

The complication rate for the TPPB in group 1 (non-experienced) was 66.1% and was 43.3% in group 2 (experienced, p<0.001). The rates of hematuria and rectal bleeding were also greater in the non-experienced group 1 (hematuria: 59.2% versus 38.3%, p<0.001; rectal bleeding: 6.3% versus 0.7%, p=0.02). All other complications were lower in the TPPB group, but without statistical significance (Table-1). Also, hematuria, rectal bleeding, and low-grade pain were statistical significantly lower for the transperineal procedure when comparing TPPB group 2 (experienced) to TRPB, (Table-1, supplementary Table-1).

Pathological Gleason and ISUP reports were similar between TPPB and TRPB groups (Table-2).

**Table 2:** Pathological results.

Pathological report	Transperineal (N=283)	Transrectal (N=513)	p value
**Gleason score – no. (%)**			0.23
**No cancer**	**119 (42.2)**	**320 (37.2)**	
6	31 (11.0)	162 (18.8)	
7 (3+4)	79 (28.0)	190 (22.1)	
7 (4+3)	27 (9.6)	107 (12.4)	
8	12 (4.3)	30 (3.5)	
9	14 (5.0)	50 (5.8)	
10	0	2 (0.2)	
**ISUP classification – no. (%)**			0.48
**No cancer**	**119 (42.2)**	**320 (37.2)**	
1	31 (11.0)	162 (18.8)	
2	79 (28.0)	190 (22.1)	
3	27 (9.6)	107 (12.4)	
4	12 (4.3)	30 (3.5)	
5	14 (5.0)	52 (6.0)	

No = number; % = percentagem

## DISCUSSION

The results of the study indicated that complication rates of TPPB declined dramatically as the team gained experience. This suggests that the learning curve is an important factor when evaluating the complications of the procedure. To date, we have not identified any studies in the literature that show the learning curve to perform TPPB.

The results of the present study favored the transperineal to transrectal approach in relation to procedure complications. All complications were reduced with this innovative technique.

Although hematuria is usually self-limited, rectal bleeding is a potentially dangerous complication. In this period, fortuitously we had no severe rectal bleeding on the TRBP group, but in the literature 2.5% TRBP presents major or moderate rectal bleeding (8). Transperineal approach theoretically eliminates this complication, once there is no need to trespass rectal mucosa, offering no risk of rectal artery lesion.

Incidence of pain related to transperineal biopsy ranges from 9.1% to 33.5% in the literature, and this complication is usually higher for this approach in relation to transrectal procedures (6, 9). All procedures were performed under anesthetic sedation or general anesthesia in this study. The anesthesia team is well experienced with interventional radiology procedures, and usually prescribes intravenous analgesic medications to optimize patient’s experience. In our study, mild pain was statistically lower in the transperineal group and pain requiring medication was lower than the TRPB group, but without statistical significance. The incidence of pain was also inferior to that the literature reports (10). The adherence of interventional radiology team to perform pudendal block and prostatic nervous plexus block with long-acting anesthetic (0.75% ropivacaine) may explain these results, added to the experienced anesthesia team.

Urinary retention was reported as a disadvantage of the transperineal technique (11). In our study, however, the rates of urinary retention were similar between the TPPB and TRPB groups, with a trend towards a lower incidence of urinary retention with the need of urinary catheterization in the postoperative period in the TPPB group.

Infectious complications are the major justification for the widespread application of transperineal biopsies (9). In accordance with literature, there were no cases of prostatitis or sepsis in the TPPB group in this study. Despite the low incidence of prostatitis in the TRPB group (0.6%), two patients had serious infections. This low incidence is probably related to the recent change in antibiotic prophylaxis (12): 2,000 mg of ceftriaxone, since 2015 was used for anesthetic induction.

The transperineal approach maintained the pathological pattern, with no statistically significant difference for the ISUP or Gleason classifications in the anatomopathological reports. Xiang et al., have not found differences in diagnostic accuracy between transperineal or transparietal techniques in a metanalysis (6). Effectively, the access route differs, but the biopsy is performed by the same basic technique: tru-cut needle and US guided with MRI software fusion.

The limitations of this study were the different experiences of the physicians when performing the two types of procedures; the diverse periods of time of patient’s enrolment; the absence of multivariable analysis considering patients characteristics; and the impossibility of pathology comparison using the two different methods in the same patient, due to ethical reasons. All the interventional radiologists had at least ten years of experience performing TRPB, some of them with poor prior experience in TPPB but still in the beginning of the learning curve. This discrepancy in experience might have skewed the results against the transperineal method, but nonetheless the results were favored for some methods. Although the groups were chosen from separate years, the time spans were temporally close, without any differences in the staff involved in the procedures or patient care afterwards. After the introduction of TPPB in the center, it became the standard of care for prostate biopsy in that institution.

Despite being a retrospective study, this research included many patients, while being the first of its kind. The results, in accordance with the international literature, demonstrate the safety of the method and quality of the professionals involved, justifying its widespread application in the near future.

## CONCLUSIONS

This study suggests that complication rates declined dramatically as the team gained experience, supporting the learning curve to perform the TPPB. The complication rate was lower in the TPPB group compared to the TRPB. Furthermore, despite being initially challenging, the targeted TPPB is safer than transrectal biopsy, offering inferior risks not only for infections, but for all types of complications, without compromising diagnostic yield. We recommend TPPB as the first choice for prostate biopsy.

## APPENDIX

**Supplementary Table 1:** - Complication rate between TPPB group 2 and TRPB.

Complication	TPPB group 2 (N=141)	TRPB (N=513)	p value
All – no. (%)	61 (43.3)	420 (81.9)	<0.001
Hematuria – no. (%)	54 (38.3)	340 (66.3)	<0.001
Rectal bleeding – no. (%)	1 (0.7)	93 (18.1)	<0.001
Pain without need of analgesia – no. (%)	4 (2.8)	48 (9.4)	0.01
Pain requiring analgesia – no. (%)	4 (2.8)	23 (4.5)	0.38
Urinary retention – no. (%)	1 (0.7)	12 (2.3)	0.32
Prostatitis – no. (%)	0	3 (0.6)	>0.99
Other – no. (%)	4 (2.8)	15 (2.9)	>0.99

TPPB = transperineal prostate biopsy; TRPB = transparietal prostate biopsy; no = number; % = percentage
